# Grip Force on Steering Wheel as a Measure of Stress

**DOI:** 10.3389/fpsyg.2021.617889

**Published:** 2021-06-07

**Authors:** Yotam Sahar, Tomer Elbaum, Michael Wagner, Oren Musicant, Tehila Hirsh, Shraga Shoval

**Affiliations:** Department of Industrial Engineering and Management, Ariel University, Ariel, Israel

**Keywords:** grip force, stress, steering wheel, driving, heart rate variability, heart rate, psychomotor tasks, physiologic indices

## Abstract

Driver performance is crucial for road safety. There is a relationship between performance and stress such that too high or too low stress levels (usually characterized by stressful or careless driving, respectively) impair driving quality. Therefore, monitoring stress levels can improve the overall performance of drivers by providing either an alert or intervention when stress levels are sub-optimal. Commonly used stress measures suffer from several shortcomings, such as time delays in indication and invasiveness of sensors. Grip force is a relatively new measure that shows promising results in measuring stress during psychomotor tasks. In driving, grip force sensor is non-invasive and transparent to the end user as drivers must continuously grip the steering wheel. The aim of the current research is to examine whether grip force can be used as a useful measure of stress in driving tasks. Twenty-one participants took part in a field experiment in which they were required to brake the vehicle in various intensities. The effects of the braking intensity on grip force, heart rate, and heart rate variability were analyzed. The results indicate a significant correlation between these three parameters. These results provide initial evidence that grip force can be used to measure stress in driving tasks. These findings may have several applications in the field of stress and driving research as well as in the vehicle safety domain.

## Introduction

“Will I have to learn how to drive?” asked the 10 year-old daughter of one of the authors recently. Although it is a common belief that autonomous cars will take over in the next few years, the more probable answer is that she would have to learn how to drive unless she is willing to rely solely on public transportation. According to Litman (2019), it is not until the 2050s that fully autonomous cars, in which no human involvement is required (known as level 5 in the autonomous driving scale defined by the Society of Automotive Engineers International), will be commonly used. Until such time, driver performance will remain critical in road safety.

Road accident investigation (Hendricks et al., 2001) and observational studies (Dingus et al., 2016) indicate that about 90% of all road accidents result from human error. To improve road safety, it is essential to recognize the factors affecting driver performance, specifically, factors that can be moderated to improve driver performance and road safety. This paper focuses on one such factor—temporal driver stress.

Stress is defined as “…a real or interpreted threat to the physiological or psychological integrity of an individual that results in physiological and/or behavioral responses” (McEwen, 2000). According to the transactional model, stress is the outcome of appraisals of demands and personal competence, together with a coping strategy that mediates between external demands (Lazarus and Folkman, 1984). Appraisal processes generate various outcomes or stress symptoms: physiological, emotional, and behavioral (Matthews, 2001). McGrath (1976) stated that stress results from an interaction between three elements: perceived demand, perceived ability to cope, and perceived importance of coping with the demand.

The concept of “stress” is often used as a synonym to the “mental workload” concept (Staal, 2004). Accordingly, the mental workload is also referred to as a transactional concept since it represents an interaction between mental capacities and task demands (Dehais et al., 2020). Furthermore, some stress definitions hold that stress represents a higher mental workload (Brookhuis and De Waard, 2010; Hou et al., 2015).

An additional definition by Mulder and Moray (1979) suggests that mental workload is “…an inferred construct that mediates between task difficulty, operator skill, and observed performance” (Mulder and Moray, 1979; p. 443). Thus, based on Mulder and Moray’s (1979) definition of mental workload and the mentioned definition of stress by McGrath (1976), the main difference between mental workload and stress stems from the perceived ability to cope with the demands, namely, unlike mental workload (Mulder and Moray, 1979), stress is caused by the perceived consequences of failing to cope with the demands (McGrath, 1976).

Indeed the confusion between the terms “workload” and “stress” is an entangled issue, as these terms are not yet adequately defined nor unambiguously differentiated in the literature. Furthermore, the manifestations through the sympathetic nervous system of stress and workload are similar and may be indistinguishable (Alsuraykh et al., 2019). This confusion will not be resolved within the framework of the present study, and henceforth we shall use only the term “stress” for simplicity.

One of the common descriptions of the relationship between performance and stress is based on findings made more than a century ago by Yerkes and Dodson (1908), later described as an “inverted U-shaped curve.” According to the inverted U-shaped curve, the upper and lower levels of stress yield unsatisfactory performance, while the mid-level produces the best performance (Hancock, 1989; Hancock and Szalma, 2008). Concerning driving, higher stress levels are harmful to driver performance (Qu et al., 2016). At the other end of the scale, very low stress, termed by Hancock and Szalma (2008) as “under-stimulation,” was found to impair driver performance (Joosen et al., 2017).

The construct of stress is divided into chronic and acute stress (Segerstrom and Miller, 2004). Chronic stress refers to a continuous state beyond a specific driving situation. Acute stress refers to a single event of short duration or a “micro-event” (Meyer et al., 2010). In the context of driving, short-duration events that may cause stress are unexpected events that, in turn, require sudden and unplanned reactions (Davies and Underwood, 2000). Stress-inducing driving events require two main maneuvers from the driver: manipulating the steering wheel and braking. Studies on driver stress have used manipulations such as driving through a labyrinth or slalom to force the driver to manipulate the steering wheel (Zontone et al., 2020) and pedestrians or other objects erupting into the road to force the driver to brake intensely (Daviaux et al., 2020).

Acute stress during driving causes a high mental workload (Wiberg et al., 2015) and adverse effects (Frasson et al., 2014) that may decrease driver performance (Brookhuis and De Waard, 2010; Rastgoo et al., 2019). Adding automation would not necessarily provide drivers with a less effortful working environment (Botzer et al., 2016). However, detecting acute stress during driving may allow various interventions that would reduce potential risks. An example of such an application is stress-adaptive car systems that modify the parameters of in-vehicle driver-aiding systems based on the driver’s stress levels (Collet and Musicant, 2019). Another application is in-car just-in-time stress management interventions (e.g., mild temperatures and music, bio-feedback interfaces, and chatbots) administered when the stress levels are too high (Balters et al., 2019).

Acute stress is manifested physiologically by the sympathetic nervous system, which stimulates the body’s “fight or flight” response. This response is antagonistic to the parasympathetic nervous system, which reduces stress (Contrada and Baum, 2011). These reactions can be measured in many ways, such as maximal heart rate (HR) (Kudielka et al., 2004), power spectra in specific frequency bands of the heart rate variability (HRV) signal (Allen et al., 2014), galvanic skin response (GSR) (Al-Fudail and Mellar, 2008), eye-related measures (Matthews et al., 2015), and cortisol levels (Yamaguchi et al., 2006).

In HRV analysis, the cardiac signal is divided into three components: VLF (very low frequency, 0–0.04 Hz), LF (low frequency, 0.04–0.15 Hz), and HF (high frequency, 0.15–0.4 Hz) (Malik, 1996). The LF measure reflects the sympathetic system (and therefore is related to stress), while the HF measure reflects the parasympathetic system (Sztajzel, 2004). The LF/HF ratio indicates the balance between the sympathetic and parasympathetic divisions of the autonomic nervous system and is used as a measure of stress as well (Kristal-Boneh et al., 1995; McCraty et al., 1995).

The measures mentioned above suffer from several practical shortcomings. Cortisol level analysis is not suitable during task performance since it is not easily measured continuously. GSR, HR, and HRV measurements may be inconvenient for use in realistic driving scenarios (Healey and Picard, 2005) and may even be considered obtrusive (Dinges et al., 2005). These measures also suffer from delays in the measurement (the time gap between the stressful event and the observed response). GSR’s delays are between 2 and 11 s long (Kucera et al., 2004; Dawson et al., 2007; Bruun, 2018), and valid analysis of changes in HRV may require a continuous signal of 4–5 min in duration (Nickel and Nachreiner, 2003). Cortisol measurement reacts to stressors with a delay of several minutes, sometimes up to half an hour (Kirschbaum and Hellhammer, 1994).

Eye-related measures, such as pupil dilation (Palinko et al., 2010), fixation duration (Matthews et al., 2015), saccade rate, and gaze shifts (Tomer et al., 2018), as well as saccadic range (May et al., 1990), were reported as indices of mental workload. There is limited evidence for ocular measures as stress indicators, and most findings are concerning pupil dilation (Pedrotti et al., 2014). Though a stressor’s administration leads to pupil dilation, the pupil’s size is susceptible to light intensity and requires an illumination-controlled environment—not practical for non-lab applications (Pedrotti et al., 2014). While eye closure level is useful in measuring drowsiness (Grimberg et al., 2020), it is also not useful for stress measurement. Finally, GSR and HRV do not always correlate strongly with stress, neither induced (e.g., Rohleder et al., 2006; Zhai and Barreto, 2006) nor measured by well-established measures, such as cortisol level (e.g., Healey and Picard, 2005).

Therefore, it may be useful to develop additional stress measures that provide solutions to the issues discussed earlier. One such possible measure is grip force, found to be capable of measuring stress in a prompt and non-invasive manner.

Continuous and repeated stress measurements using non-invasive methods have been of great interest in recent years. Hernandez et al. (2014) measured the amount of force applied to a computer keyboard and a mouse. Although not suitable for use in driving tasks, it shows that hand muscle tonus measurement has the potential to be an indication of stress. Wahlström et al. (2002) examined the effect of stressors (e.g., time pressure and verbal provocation) on various factors, including the grip force upon a computer mouse. Grip force increased when stressors were used. However, this effect was attributed both to stress and the mouse operation’s speed. In another research that used grip force on a computer mouse, Liao et al. (2006) found greater grip force in response to higher time pressure. It should be noted that these studies manipulated the mental workload rather than the stress, as no direct implications for low-performance outcomes were involved. These two studies also used static tasks (e.g., math problems and typing tasks), making it difficult to extend their findings to other contexts such as driving tasks.

Wagner et al. (2015) examined the feasibility of grip force as a measure of stress in tracking tasks. Grip force was higher in the presence of stress. This study provides initial evidence of distinguishing between stressful and non-stressful conditions during physical tracking tasks by measuring the grip force. Recently, these findings were successfully reconstructed (Botzer et al., 2020). Mühlbacher-Karrer et al. (2017) used the measurement of a driver’s grip force on a steering wheel as part of a stress estimation system. However, the grip force’s contribution to the stress level calculation was only 10%. Another limitation of this study is that it was conducted only in a simulator and not in actual driving, ignoring the effect of other factors on grip force other than stress (e.g., vehicle accelerations). Additionally, the mere driving an actual car in an experiment is known to induce a state of stress (Balters et al., 2019), as the consequences of one’s performance are tangible, unlike participating in an experiment conducted in a simulator.

We aim to study the relationship between grip force and other more common indices of stress, namely, physiological indices and performance indices during short-duration driving events. The purpose of the study, hopefully its main contribution, is to provide initial empirical evidence to grip force as an index of stress in driving tasks. To this end, one should define the appropriate driving events that elicit acute stress. In previous research, forced changes in driving behavior were found to cause stress (Ross and Burnett, 2001; Lee and Winston, 2016; Saxena, 2017). Such changes may result from the road conditions and unexpected factors that force emergency maneuvers (i.e., a braking sign or a figure bursting into the road). Specifically, stopping in response to a STOP sign during driving and the necessity to brake have been found to induce stress in an experimental context (Min et al., 2002; Collet et al., 2014; Prasolenko et al., 2017; Sugiono et al., 2019).

Thus, in this study, we used diverse heart rate measurements to show that braking events lead to higher stress as manifested in psychophysiological changes of heart measurements. By manipulating stress-inducing driving events and measuring their effect on the heart and also grip force measurements, we aim to verify the use of grip force as a valid measure of stress during driving.

In the current research, grip force and heart rate measures (HRV and HR) were recorded while driving and braking in various intensities (in response to a STOP sign), as described in “Experimental Setup and Methods.” We hypothesized that intense braking during driving affects grip force and elicits correlations between grip force, HRV, and HR measures. Thus, grip force data were analyzed as a function of braking intensity, and correlations among grip force and heart rate measures (HRV and HR) were calculated to validate the grip force measure against an accepted measure of stress (see section “Results”). HRV and HR data were also analyzed as a function of braking intensity, serving as a manipulation check.

## Experimental Setup and Methods

### Participants

Twenty-one participants took part in this study. Due to technical issues (failure in recording the data of one participant), we used the data from 20 participants. All participants were bachelor course students. All participants were male, between the ages of 24 and 34 (average 28.45, SD 2.18), and had a private car driver’s license for at least 4 years.

Before the experiment, the participants underwent a safety briefing, including a description of the experimental task, and completed an informed consent statement. A safety supervisor, positioned in the front passenger seat, was responsible for maintaining safety during the experiment.

### Apparatus

**FIGURE 1:**
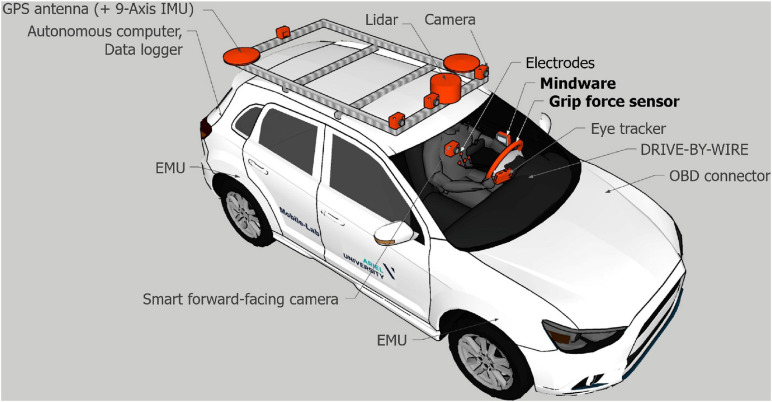
The Mobile Lab, equipped with GPS and sensors, Mindware and grip force sensors, as well as additional equipment that was not used in the current research.

The Mobile-Lab^1^ is equipped with sensors for monitoring the vehicle and road environment, including inertial measurement units (Lidar, GPS antennas, and several cameras), and sensors for monitoring indices of the driver using the Mindware Mobile Impedance Cardiograph (heart activity measurement system Model 50–2303-00, 2014, with a sampling rate of 500 Hz and 24-bit ADC digitization). Cardiac data were recorded from electrodes affixed to the participant’s chest. A grip force self-developed measurement system was used (utilizing a force-sensitive resistor sensor, sampled by an Arduino UNO R3 board). Both systems were equipped with three-axis accelerometers. The experiment was conducted using an instrumented Kia Nero (hereafter, the “Mobile-Lab” Figure 1).

### Procedure

**FIGURE 2:**
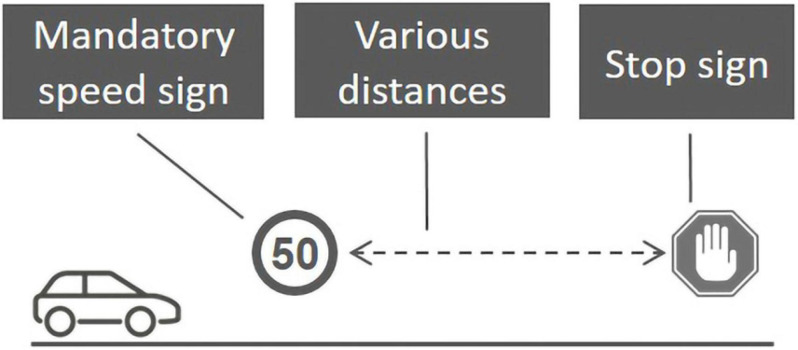
The general layout of the experimental driving session. Experimental manipulations: mandatory speed sign (one of two speeds: 50 or 60 km/h) and STOP sign (at various distances: 15, 20, 25, 30, 35, or 40 m). Each participant performed the driving sessions under all combinations of speeds and braking distances at random order.

The participants performed 12 experimental driving sessions. Each session involved driving along a straight path of approximately 200 m at one of two mandatory speeds (50 or 60 km/h) and braking at varied mandatory distances (15, 20, 25, 30, 35, or 40 m), as shown in Figure 2. Each participant performed the driving sessions under all combinations of speed and braking distances (two speeds × six distances = 12 conditions) in random order. Each participant performed two training sessions of about 2 min (during which they got acquainted with the experimental path) and 12 experimental driving sessions (one for each condition), which lasted nearly 20 min overall. After each experimental driving session, the participant was instructed to leave the vehicle stationary for 15 s.

### Data Preparation and Analysis Procedures

**FIGURE 3:**
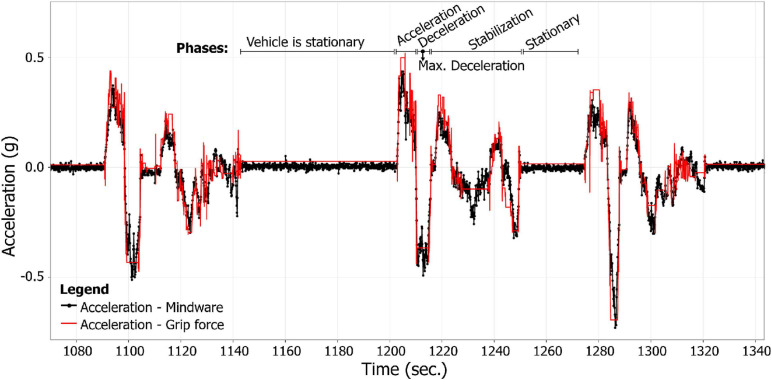
Synchronized acceleration vectors of both accelerometers (of the Mindware system—in black—and the self-developed grip force measurement system—in red) in the direction of the vehicle’s travel, during three successive experimental driving sessions, of a single participant.

**FIGURE 4:**
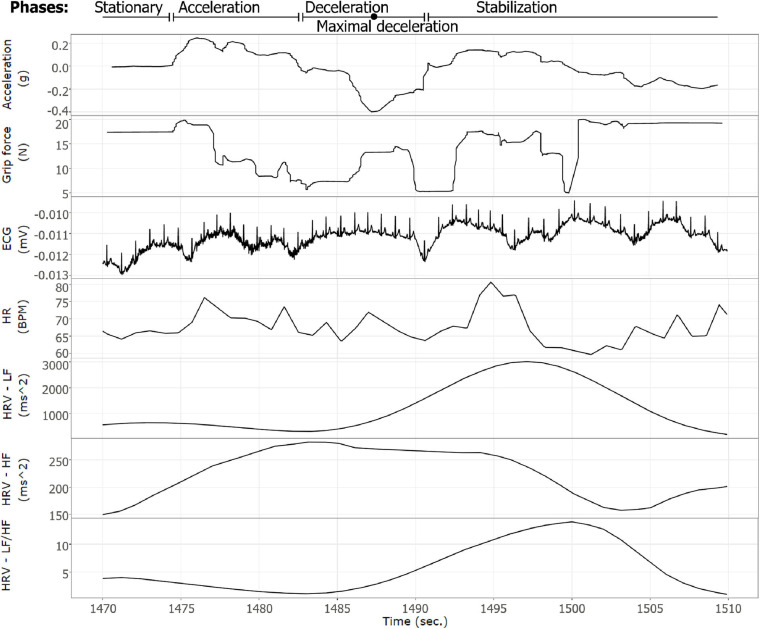
An example of a braking event as expressed by the various measures recorded. A single braking event of participant number 1. The *X*-axis represents time (s), plots (from top to bottom): acceleration (g), grip force (N), raw ECG signal (mV), HR (BPM), HRV—LF (ms^2^), HRV—HF (ms^2^), and HRV—LF/HF ratio.

**FIGURE 5:**
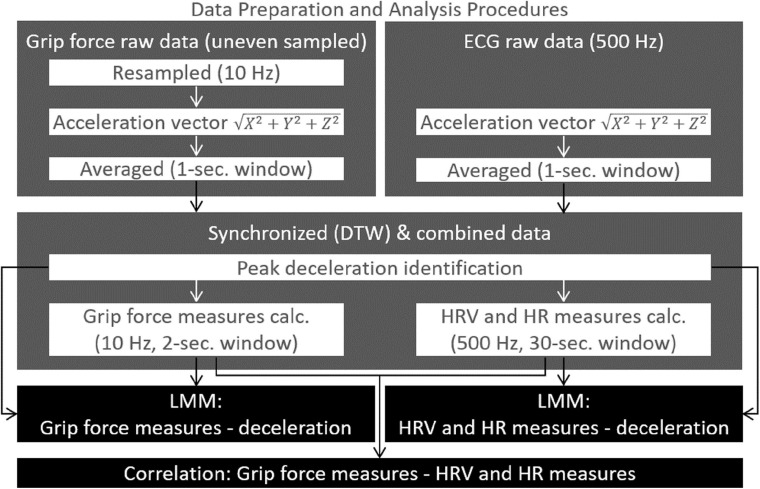
Data preparation and analysis procedures: a workflow description of the data preparation procedures, data synchronization procedure, and the data analyses used.

To analyze the data from both acquisition systems used (Mindware and the self-developed grip force measurement system), first we synchronized the data (see section “Data Synchronization”). After the data synchronization, we used the acceleration data to identify the peak deceleration for each driving session. Each session was characterized by a static phase (of at least 15 s), an acceleration phase, and a deceleration phase (as demonstrated in Figures 3, 4), in contrast to other acceleration data (e.g., data from the training sessions) which were less organized. Later, HRV and HR heart rate measures and grip force measures were calculated (see “Heart Rate Measures Calculation” and “Grip Force Data Preparation and Calculation”) and analyzed (see section “Data Analysis”). Figure 5 summarizes the flow of these processes.

#### Data Synchronization

Cardiac activity data and grip force data were synchronized *post factum*, according to the accelerometers’ data (from both measuring systems), using a dynamic time wrapping (DTW) algorithm. Both data acquisition systems (Mindware for HRV data and the self-developed grip force measurement system) were equipped with three-axis accelerometers (X,Y, and Z), which were fixed to the vehicle’s chassis. Acceleration data were recorded by each system in a synchronized manner with the physiological data (HRV and HR data at the Mindware system and grip force data at the grip force measurement system).

First, the grip force system’s sampling rate was uneven and ranged between 80 and 120 ms (8–12 Hz). Since a pre-condition of the DTW procedure is that “the data should be sampled at equidistant points in time” (Senin, 2008), a standard method to deal with this requirement is resampling the data as has been done in the current study. The resampled grip force data had a 10 Hz sampling rate. The Mindware system had a sampling rate of 500 Hz.

Next, for each system separately, a unified vector of the three axes was calculated (${}_{\sqrt{X^{2}+Y^{2}+Z^{2}}}$), using a 1-s sliding window. Finally, a DTW algorithm was used to synchronize these acceleration vectors from both systems. A similar synchronization method has been used by Mantilla et al. (2017) to detect temporal synchronization. DTW was proven to be a robust distance measure for time series, enabling the matching of similar plots even if they are out of phase in the time axis (Keogh and Ratanamahatana, 2005).

#### Heart Rate Measures Calculation

To properly calculate the HRV LF measure and LF/HF ratio, a minimum sliding window size of 30 s is required (De Rivecourt et al., 2008; Wang et al., 2009). Typically, an HRV window size is between 20 s and several minutes. For example, Mulder et al. (2009) used a 300-s window, whereas Healey and Picard (2005) used 100- and 300-s windows. It should be noted that the decision about the window size is often arbitrary.

A small window size of 30 s is sufficient if combined with a short-time Fourier transform (Li et al., 2019). Therefore, in the current research, a sliding window with a window size of 30 s was implemented to the cardiac data for computing the HRV LF and HF measures and the LF/HF ratio as well as for heart rate. The center of the window was determined according to the braking event’s maximal deceleration. Since there were pauses of 15 s after each braking event (as described in “Procedure”), there could not have been any other experimental effect on the physiological signals during the entire window size other than the effect of the forced braking event itself. In addition, to account for the chi-square distribution of HRV and heart rate values (Van Roon et al., 2004), a natural log transformation was applied to these measures.

#### Grip Force Data Preparation and Calculation

Grip force data under the threshold of 5 N (newton) was considered mostly white noise due to its proximity to the lower boundary of the grip force sensitivity. Accordingly, grip force data below 5 N was excluded. Grip force data was collected and resampled at a 10 Hz rate (as detailed in “Data Synchronization”). Grip force measures were calculated to explore various aspects of grip force in relation to the other measures. The grip force measures calculated were mean, maximum (max), and standard deviation (sd).

These grip force measures were calculated using a 2-s time window, centered around each braking event’s peak deceleration. Due to this study’s preliminary nature, there are no widely accepted guidelines to rely on for grip force window size in stress measurement. In defining the time window, we referred to the preliminary findings from an ongoing study regarding this issue, which shows an initial inclination toward the use of a narrow time window of fewer than 5 s in calculating grip force as a measurement of stress (Botzer et al., 2020). Additionally, we based this decision on the indication received from an analysis detailed and illustrated in Appendix A.

Based on psychophysical and physiological reports and stress models (Liu and Ulrich, 2014), exponential logarithmic or sigmoid transformation functions, rather than linear functions, are expected. For example, electromyography is reported to have a logarithmic transfer function (Rezazadeh et al., 2012). Therefore, a natural log transformation was applied to the grip force calculated measures since the higher end of grip force is limited by the maximal grip strength (for each individual).

#### Data Analysis

The linear mixed model (LMM) was selected to analyze the effects of braking intensity on the various physiological measures, primarily due to its suitability to repeated-measures designs (Peat and Barton, 2014). In this method, within-subject correlations are modeled using the covariance structure, built on the variance around the outcome measurement at each time point and on the correlations between measurements taken at different times from the same participant (Peat and Barton, 2014).

A meta-analysis method was used to analyze the correlation of grip force measures with HR and HRV measures. The correlations between these measures for each participant served as the meta-analysis input. This procedure enabled the consideration of inter-personal variance (for further description, see “Results”).

## Results

**FIGURE 6:**
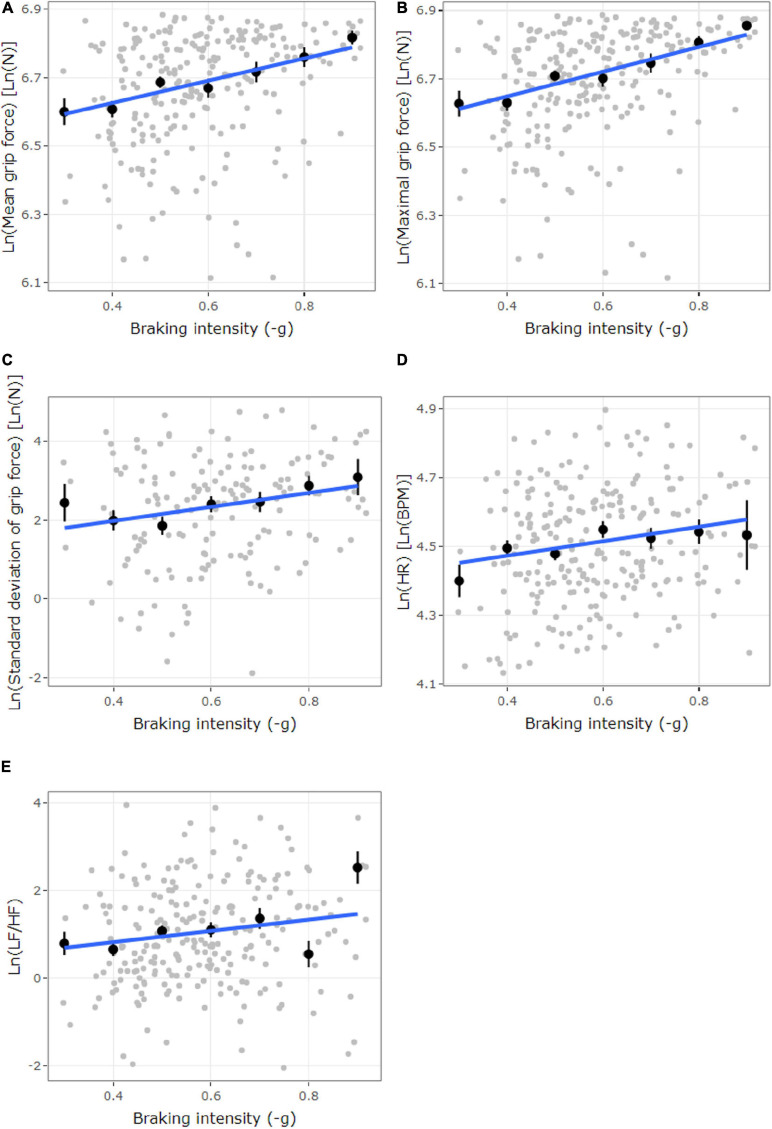
The plots illustrate the significant LMM models described in Table 1. Higher *x*-axis values (-g) represent a higher braking intensity in this figure. **(A–C)** Ln-transformed grip force [Ln(N)], **(A)** mean grip force, **(B)** maximal grip force, and **(C)** standard deviation of grip force, as a function of braking intensity (–g). **(D)** Ln-transformed HR [Ln(BPM)] as a function of braking intensity (–g). **(E)** Ln-transformed HRV LF/HF as a function of braking intensity (–g). Gray dots represent observations. Black dots represent the mean of each binned group of observations (according to deceleration), with 95% confidence interval. The blue line represents smoothed conditional means using lm smoothing.

To examine our hypothesis that compelled braking during driving elicits correlating measured patterns of grip force and heart rate, we first explored the braking events’ effects on grip force. All three LMMs were fitted to the data with the assumption of a linear relationship in order to study the nature of the relationship between the three grip force measures (i.e., Ln transformation of mean, max, and sd of grip force) and the *D* parameter for maximal deceleration (i.e., the intensity of braking events). Maximal deceleration (D) was included in the model as the predictor and grip force measures as the predicted variables (see rows 1–3 in Table 1 for a formal description of these three-mixed effect models and Figures 6A–C for their visual depiction).

**TABLE 1:** Summary of linear mixed model analyses for various models.

Model	β_0_ (SE)	β_1_ (SE)	*F*	*P*	Adj. *R*^2^
**Grip force**					
*Ln*(*meanGF*) = β_0_ + β_1_(*D*) + *b*_*i*_ + ε	6.527 (0.044)	0.244 (0.061)	16.4	0.0001***	0.465
*Ln*(*maxGF*) = β_0_ + β_1_(*D*) + *b*_*i*_ + ε	6.512 (0.042)	0.318 (0.061)	26.54	< 0.0001***	0.375
*Ln*(*sdGF*) = β_0_ + β_1_(*D*) + *b*_*i*_ + ε	−6.724 (1.182)	10.401 (1.896)	30.963	< 0.0001***	0.209
**HR and HRV**					
*Ln*(*LF*/*HF*) = β_0_ + β_1_(*D*) + *b*_*i*_ + ε	1.333 (0.235)	0.21 (0.37)	3.33308	0.0693⋅	0.19
*Ln*(*LF*) = β_0_ + β_1_(*D*) + *b*_*i*_ + ε	5.852 (0.257)	−0.15 (0.354)	1.2628	0.2624	0.453
*Ln*(*HR*) = β_0_ + β_1_(*D*) + *b*_*i*_ + ε	4.438 (0.038)	0.097 (0.025)	41.89	< 0.0001***	0.882
*Ln*(*HF*) = β_0_ + β_1_(*D*) + *b*_*i*_ + ε	4.546 (0.264)	−0.262 (0.331)^*a*^	0.6404	0.4244	0.51

mean GF, mean grip force; maxGF, maximum grip force; sdGF, standard deviation of the grip force; LF, HF, and LF/HF, heart rate variability measures; HR, heart rate, D, maximal deceleration (−g) during the braking event; b_*i*_, random effect parameter for driver i; ε, error term. *^*a*^HRV HF’s negative value represents the inhibitory pattern of the parasympathetic system during stressful situations. It is assumed that b_*i*_ ∼ N(0,σ_*b*_).* Significance codes: 0 ≤ “***” < 0.001 < “**” < 0.01 < “*” < 0.05 < “.” < 0.1 < “” ≤ 1.

Significant main effects were found for maximal deceleration on all three grip force transformations (*p* < 0.001; see rows 1–3 in Table 1). Based on the models’ coefficients (rows 1–3 in Table 1 and Figures 6A–C) and in accordance with our hypothesis, the findings indicate that higher deceleration (braking intensity) predicted greater grip force (i.e., mean and maximum grip force) and larger changes in grip force (i.e., grip force sd).

An additional four-LMM analysis was performed to investigate whether braking events elicit stress as manifested by HRV and HR measures (the models are described in rows 4–7 of Table 1). The four additional LMMs include the *D* parameter for maximal deceleration (i.e., braking intensity) as the predictor and Ln-transformed HRV (LF, HF, and LF/HF ratio) and HR measures as predicted variables (see rows 4–7 in Table 1 for a formal description of these three mixed-effect models).

The additional LMM analysis showed a main effect for maximal deceleration on heart rate (*p* < 0.001; row 6 in Table 1 and Figure 6D), and a moderate trend toward significance was also found on HRV LF/HF ratio (*p* = 0.069; row 4 in Table 1 and Figure 6E). The LMMs for maximal deceleration on HRV LF and HF were not significant (*p* = 0.262 and *p* = 0.424, respectively). Based on the additional models’ coefficients for maximal deceleration on HRV LF/HF ratio and HR (rows 4 and 6 in Table 1 and Figures 6D,E) and in accordance with the assertion presented in the introduction (i.e., that braking events induce stress), higher declaration (braking intensity) predicted greater HRV LF/HF ratio and HR.

Our hypothesis addressed the association between grip force and HRV and HR as prevalent measures of stress. Specifically, the hypothesis aimed to serve as an additional association between grip force and stress. To test this hypothesis, Pearson correlations were calculated separately for each participant, followed by a meta-analysis procedure. This integration of the two procedures (i.e., separate correlations followed by a meta-analysis) was designed to include the participants as a random effect, partly similar to using LMM. Separate correlations were conducted between heart measures (HRV and HR) and the grip force’s central tendency indices (i.e., mean and maximum grip force) as mentioned above.

The meta-analysis procedure was applied for the separate correlations, using the “meta” R package. The meta-analyses examined all possible correlations between each heart measure and each grip force measure. The effect sizes were transformed into standard values using Fisher’s *r* to *z* transformation (Rosenthal, 1991). The *z*-transformed score has a standard error of 1(n−3), where *n* is the number of braking events for each participant. The inverse of this error was used as a weight for each individual *z*-transformed score so that participants with smaller standard errors were given more emphasis. After this weighting, all participants’ values were aggregated by averaging their *z*-transformed scores. Rosenthal (1991) suggests this as a conservative procedure. Finally, *z*-transformed scores were translated back to *r* values. The meta-analysis was applied to the grip force’s central tendency indices and heart rate measures (HRV LF, HRV HF, HRV LF/HF ratio, and HR). Additionally, natural log transformation was used for all heart rate and grip force measures. Table 2 contains the descriptions of the correlations and the values of the meta-analysis’ coefficients.

**TABLE 2:** Summary of meta-analyses of correlations, for all *k* = 20.

	Measures in correlation	Pearson’s *r*	Fisher’s *z*	*p*	95% CI
1.	Ln(meanGF)–Ln(LF/HF)	0.1108	7.66	< 0.0001***	[0.0826, 0.1388]
2.	Ln(maxGF)–Ln(LF/HF)	0.0622	4.29	< 0.0001***	[0.0338, 0.0905]
3.	Ln(meanGF)—Ln(LF)	−0.0513	−3.53	0.0004***	[−0.0796, −0.0229]
4.	Ln(maxGF)–Ln(LF)	−0.002	−0.14	0.8921	[−0.0304, 0.0265]
5.	Ln(meanGF)–Ln(HR)	0.0293	2.02	0.0436*	[0.0008, 0.0577]
6.	Ln(maxGF)–Ln(HR)	0.0395	2.72	0.0065**	[0.0110, 0.0679]
**HRV HF negative values represent the inhibitory pattern of the parasympathetic system during stressful situations**					
7.	Ln(meanGF)–Ln(HF)	−0.1631	−11.33	< 0.0001***	[−0.1907, −0.1353]
8.	Ln(maxGF)–Ln(HF)	−0.09	−6.21	< 0.0001***	[−0.1181, −0.0617]

meanGF, mean grip force; maxGF, maximum grip force; sdGF, standard deviation of the grip force; LF, HF, and LF/HF, heart rate variability measures; HR, heart rate. Significance codes: 0 ≤ “***” < 0.001 < “**” < 0.01 < “*” < 0.05 < “.” < 0.1 < “” ≤ 1.

Six of the eight correlations were significant, and the correlations’ direction was consistent with our hypothesis (i.e., significant positive correlations for grip force with HRV LF/HF ratio and with HR; significant negative correlations for grip force with HRV HF negative values represent the inhibitory pattern of the parasympathetic system during stressful situations). The direction of the correlation between mean grip force and HRV LF was not consistent with the hypothesis, and the correlation between max grip force and HRV LF was not significant (*p* = 0.892). Although four of the six significant correlations were highly significant (*p* < 0.001), their effect size was relatively small (i.e., smaller than 0.3, Cohen, 1992).

## Discussion

This study’s main aim was to examine the feasibility of detecting driver stress by grip force measurement in actual driving scenarios. Accordingly, the study’s main goal of indicating that grip force can serve as a measure of stress in driving tasks was mostly achieved.

The assertion that braking as a response to a STOP sign elicits stress has a vast support (e.g., Min et al., 2002; Collet et al., 2014; Prasolenko et al., 2017; Sugiono et al., 2019). Accordingly, the LMM analyses conducted in the current study revealed that, during braking as a response to a STOP sign, maximal deceleration had a highly significant effect on the HR measure. In addition, a moderate trend toward significance was found regarding maximal deceleration’s effect on HRV LF/HF ratio measure. Since these measures (HRV LF/HF ratio and HR) are referred to as stress measures (Kristal-Boneh et al., 1995; McCraty et al., 1995; Sztajzel, 2004; Allen et al., 2014), this finding offers additional support for braking as a stress-inducing driving event.

Braking intensity had a highly significant effect on all grip force measures. This finding, combined with the re-confirmed assertion that braking events induce stress, leads to a possible deduction that grip force constitutes an indication of stress. However, unlike HR and HRV, grip force may also be affected during braking by the task itself. Therefore, another possible explanation for consideration is that, during braking events, grip force may have been affected by the braking task solely or by a joint effect of stress and the braking task.

The analyses also showed correlations of HRV HF and LF/HF ratio with grip force’s transformations. The correlations of HRV HF with grip force’s transformations had a negative direction, consistent with the parasympathetic system’s inhibitory pattern during stressful situations (Hall et al., 2004; Hjortskov et al., 2004; Vuksanović and Gal, 2007). The correlations of HRV LF/HF ratio with grip force’s transformations had a positive direction as can be expected since HRV LF/HF ratio is known to increase during stressful situations. These findings may serve as further modest validation of grip force as a measure of stress. It should be noted that these correlations were weak. However, this may result from the different acquisition systems used (Milstein and Gordon, 2020). Additionally, weak correlations among different physiological measures of mental states are not an unfamiliar phenomenon (Contrada and Baum, 2011).

In this study, the participants had to brake in response to a STOP sign, which is known to induce stress, a response that was also found here as expressed by the effect of braking intensity on HR and on HRV LF/HF ratio. Grip force’s magnitude was also affected by the intensity of these braking events, a finding that lends partial support to our hypothesis that grip force is an indication of stress during driving events. This hypothesis received further support by the correlations of grip force and HR and HRV measures. Therefore, it is feasible that grip force can be used as a measure of stress in braking events during actual driving. These findings may contribute to further investigations needed to establish this relatively new measure of stress, specifically in driving contexts.

As found in the current study, a stressor’s physiological response during driving can be detected using grip force upon a steering wheel, even with a small time window of 2 s. Compared to other more established measures of stress, such as HRV, which require much larger time windows, grip force’s narrow window can enable a “real-time” assessment of the effect of stressful situations on the driver.

According to the summation of the current research findings, grip force may be considered as one of the measures of stress in mobile environments such as vehicles. By measuring the driver’s stress level in “real time,” various interventions can be employed to prevent calamities from occurring due to inferior human performance under stressful conditions. The usage of non-invasive measures of stress that do not interfere with the user’s experience allows access to the information in the realistic environment of vehicles despite the limitations inherent in it.

Measuring stress in a vehicle is beneficial not only for human-controlled cars but also for self-driving vehicles. It is clear that, in such scenarios, the grip force measurement will not be on the steering wheel but at different grip points in the vehicle or on mobile devices held by the passengers, such as smartphones and tablets. Information about the passengers’ stress levels may aid the vehicle’s control system to adjust its conduct to minimize stress and thus achieve a better user experience. Moreover, by measuring grip force exerted on a surface of a non-operation means, stress measurement may reflect a purer indication of stress, without possible influences of task-conducting-related grip force.

The current research has some limitations. First, due to the difficulty to differentiate stress from related terms (Alsuraykh et al., 2019), it should be mentioned that the manipulation used in the current study (i.e., a STOP sign as a mandatory stopping position) may not have been experienced solely as stress by the participants. Second, this study examined a limited range of potential stress-eliciting driving events. Additional driving events such as lane crossing, overtaking, or driving in heavy traffic should also be evaluated in a similar manner to gain more comprehensive insights regarding the potential of driving incidents being used as stressors. This may also help clarify whether grip force was a result of stress elicited during braking.

The third limitation is the use of a homogeneous population, constituted of male students only, with a limited range of ages. This uniform sample limits this research’s external validity, and further research should be conducted using more heterogenic samples. Finally, the “noisy” signal of the measured grip force (as illustrated in Figure 4) may interfere with the analysis of the state of the driver. Therefore, other data processing methods (e.g., fast Fourier transform) should be considered if real-time assessment is required (Zak et al., 2020).

## Conclusion

The current research’s primary purpose and contribution are to provide initial empirical evidence on the extent to which grip force may serve as an additional index of stress in driving tasks and its validation using HR and HRV measures. Heart measures were affected by braking, a finding which is consistent with the findings of previous studies and which establishes the assertion that braking events induce stress. Variations in grip force as an outcome of these stress-inducing braking events support its suitability for stress measurement in driving scenarios. The correlation of grip force and heart measures strengthens the statement that, similar to heart measures, grip force is an appropriate measure of stress.

The ability to identify a specific change in stress during a driving scenario using a non-invasive measurement tool which is transparent to the end user has the potential of introducing in-car just-in-time stress management interventions. It may also help develop a stress-adaptive car system that may adjust its conduct according to the driver’s current level of stress. Future investigations may aid in describing the relationship of grip force and stress in driving as well as in other tasks.

## Data Availability Statement

The raw data supporting the conclusions of this article will be made available by the authors, without undue reservation.

## Ethics Statement

The studies involving human participants were reviewed and approved by the Ariel University Human Ethics Committee. The patients/participants provided their written informed consent to participate in this study.

## Author Contributions

YS with the guidance of MW and SS, conceptualized the study and chose the theoretical framework. YS, OM, TE, and TH designed and conducted the experiment, chose the methods, and collected the data. OM, TE, and TH obtained the ethics committee’s approval. YS, OM, and TE analyzed the data. YS wrote the manuscript. MW, SS, OM, and TE read and revised the manuscript several times. All authors contributed to the article and approved the submitted version.

## Conflict of Interest

The authors declare that the research was conducted in the absence of any commercial or financial relationships that could be construed as a potential conflict of interest.

## Figures and Tables

**FIGURE A1:**
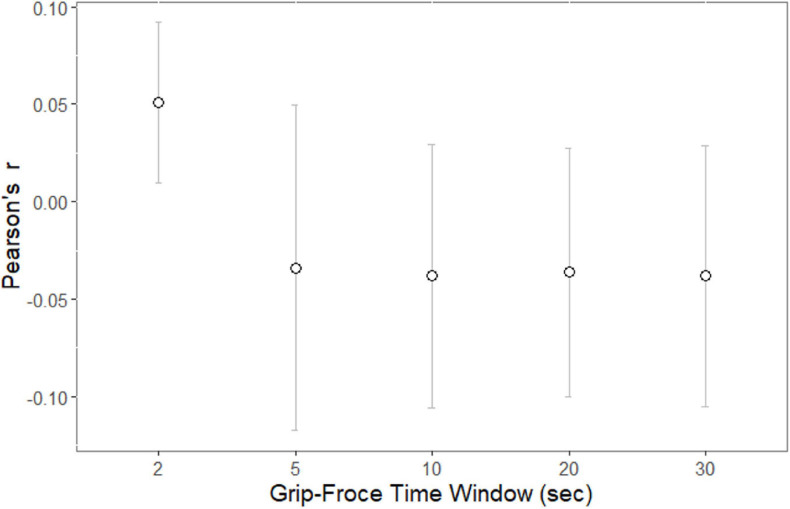
Confidence intervals of the Pearson correlation coefficient (*r*) for meta-analyses of Ln-transformed grip force’s measures (mean, maximum, and standard deviation) with Ln-transformed heart rate measures (HRV LF, HRV HF, HRV LF/HF ratio, and heart rate) for each grip force’s time window (2, 5, 10, 20, and 30 s).

## Footnotes

1 http://www.ariel.ac.il/wp/mobile-lab/

## Appendix A—Defining Grip Force’s Time Window

To define the proper time window in the calculation of the grip force measures, we explored the following time windows: 2, 5, 10, 20, and 30 s. We have conducted the meta-analysis procedure described before (as detailed in the “Data Analysis” and “Results” sections) for each of these time windows.

For each combination of the three Ln-transformed grip force transformations (mean, maximum, and standard deviation) and the four Ln-transformed heart rate measures (HRV LF, HRV HF, HRV LF/HF ratio, and HR), we have calculated the Pearson correlation coefficients.

The following plot (see Figure A1) represents the confidence intervals of the Pearson correlation coefficients for each of these time windows.

As indicated in the plot, for the 2-s time window, the lower end of the confidence interval is larger than zero. From this, it seems that the choice of the 2-s time window is reasonable.

## References

[B1] Al-Fudail M. Mellar H. (2008). Investigating teacher stress when using technology. *Comput. Educat*. 51 1103–1110. 10.1016/j.compedu.2007.11.004

[B2] Allen A. P. Kennedy P. J. Cryan J. F. Dinan T. G. Clarke G. (2014). Biological and psychological markers of stress in humans: focus on the Trier Social Stress Test. *Neurosci. Biobehav. Rev*. 38 94–124. 10.1016/j.neubiorev.2013.11.005 24239854

[B3] Alsuraykh N. H. Wilson M. L. Tennent P. Sharples S. (2019). “How stress and mental workload are connected,” in *Proceedings of the 13th EAI International Conference on Pervasive Computing Technologies for Healthcare*, (New York: Association for Computing Machinery), 371–376.

[B4] Balters S. Bernstein M. Paredes P. E. (2019). “On-road stress analysis for in-car interventions during the commute,” in *Extended Abstracts of the 2019 CHI Conference on Human Factors in Computing Systems*, (New York: Association for Computing Machinery).

[B5] Botzer A. Meyer J. Parmet Y. (2016). Effects of cues in a binary categorization task on dual-task performance, mental workload, and effort. *J. Exp. Psychol.* 22:350. 10.1037/xap0000095 27505049

[B6] Botzer A. Sahar Y. Wagner M. Elbaum T. (2020). ^∗^*Analyzing Individuals’ Grip Force Over Short Intervals in a Joystick-Controlled Task with and without a Stress Manipulation. Manuscript submitted for publication.*

[B7] Brookhuis K. A. De Waard D. (2010). Monitoring drivers’ mental workload in driving simulators using physiological measures. *Accid. Anal. Prev.* 42 898–903. 10.1016/j.aap.2009.06.001 20380918

[B8] Bruun A. (2018). “It’s not complicated: a study of non-specialists analyzing GSR sensor data to detect UX related events,” in *Proceedings of the 10th Nordic Conference on Human-Computer Interaction*, (New York: Association for Computing Machinery), 170–183.

[B9] Cohen J. (1992). “Things I have learned (so far),” in *Annual Convention of the American Psychological Association, 98th, Aug, 1990, Boston, MA, US; Presented at the aforementioned conference*, (Washington, DC: American Psychological Association).

[B10] Collet C. Musicant O. (2019). Associating vehicles automation with drivers functional state assessment systems: a challenge for road safety in the future. *Front. Hum. Neurosci.* 13:131. 10.3389/fnhum.2019.00131 31114489 PMC6503868

[B11] Collet C. Salvia E. Petit-Boulanger C. (2014). Measuring workload with electrodermal activity during common braking actions. *Ergonomics* 57 886–896. 10.1080/00140139.2014.899627 24689861

[B12] Contrada R. J. Baum A. eds (2011). *The Handbook of Stress Science: Biology, Psychology, and Health.* Berlin: Springer.

[B13] Daviaux Y. Bonhomme E. Ivers H. de Sevin É. Micoulaud-Franchi J. A. Bioulac S. Altena E. (2020). Event-Related Electrodermal Response to Stress: results From a Realistic Driving Simulator Scenario. *Hum. Fact.* 62 138–151. 10.1177/0018720819842779 31050918

[B14] Davies M. N. O. Underwood G. (2000). Cognition and stress. *Encycl. Stress* 1 478–483.

[B15] Dawson M. E. Schell A. M. Filion D. L. (2007). The electrodermal system. *Handb. Psychophysiol*. 2 200–223.

[B16] De Rivecourt M. Kuperus M. N. Post W. J. Mulder L. J. M. (2008). Cardiovascular and eye activity measures as indices for momentary changes in mental effort during simulated flight. *Ergonomics*, 51 1295–1319. 10.1080/00140130802120267 18802817

[B17] Dehais F. Lafont A. Roy R. Fairclough S. (2020). A Neuroergonomics Approach to Mental Workload, Engagement and Human Performance. *Front. Neurosci*. 14:268. 10.3389/fnins.2020.00268 32317914 PMC7154497

[B18] Dinges D. F. Rider R. L. Dorrian J. McGlinchey E. L. Rogers N. L. Cizman Z. Metaxas D. N. (2005). Optical computer recognition of facial expressions associated with stress induced by performance demands. *Aviat. Space Environ. Med*. 76 B172–B182. 15943210

[B19] Dingus T. A. Guo F. Lee S. Antin J. F. Perez M. Buchanan-King M. Hankey J. (2016). Driver crash risk factors and prevalence evaluation using naturalistic driving data. *Proc. Natl. Acad. Sci*. 113 2636–2641. 10.1073/pnas.1513271113 26903657 PMC4790996

[B20] Frasson C. Brosseau P. O. Tran T. H. D. (2014). “Virtual environment for monitoring emotional behaviour in driving,” in *International Conference on Intelligent Tutoring Systems.* eds Trausan-Matu S. Boyer K.E. Crosby M. Panourgia K. (Cham: Springer), 75–83 10.1007/978-3-319-07221-0_10

[B21] Grimberg E. Botzer A. Musicant O. (2020). Smartphones vs. in-vehicle data acquisition systems as tools for naturalistic driving studies: a comparative review. *Safe. Sci.* 131:104917. 10.1016/j.ssci.2020.104917

[B22] Hall M. Vasko R. Buysse D. Ombao H. Chen Q. Cashmere J. D. Thayer J. F. (2004). Acute stress affects heart rate variability during sleep. *Psychos. Med.* 66 56–62. 10.1097/01.psy.0000106884.58744.09 14747638

[B23] Hancock P. A. (1989). A dynamic model of stress and sustained attention. *Hum. Fact.* 31 519–537. 10.1177/001872088903100503 2625347

[B24] Hancock P. A. Szalma J. L. (Eds.). (2008). *Performance under stress.* Farnham: Ashgate Publishing Ltd.

[B25] Healey J. Picard R. W. (2005). Detecting stress during real-world driving tasks using physiological sensors. *IEEE Trans. Intell. Transp. Syst.* 6 156–166. 10.1109/tits.2005.848368

[B26] Hendricks D. L. Freedman M. Fell J. C. (2001). *The Relative frequency of Unsafe Driving Acts in Serious Traffic Crashes (No. DOT-HS-809-206).* Washington, DC: National Highway Traffic Safety Administration.

[B27] Hernandez J. Paredes P. Roseway A. Czerwinski M. (2014). “Under Pressure: Sensing Stress of Computer Users,” in *Proceedings of the SIGCHI conference on Human factors in computing systems*, (New York: ACM), 51–60.

[B28] Hjortskov N. Rissén D. Blangsted A. K. Fallentin N. Lundberg U. Søgaard K. (2004). The effect of mental stress on heart rate variability and blood pressure during computer work. *Eur. J. Appl. Physiol.* 92 84–89. 10.1007/s00421-004-1055-z 14991326

[B29] Hou X. Liu Y. Sourina O. Mueller-Wittig W. (2015). “CogniMeter: EEG-based emotion, mental workload and stress visual monitoring,” in *2015 International Conference on Cyberworlds (CW)*, (New York: IEEE), 153–160

[B30] Joosen P. Exadaktylos V. Taelman J. Berckmans D. (2017). “The effect of Individual Stress Zones on car-racing performance,” in *2017 IEEE 14th International Conference on Wearable and Implantable Body Sensor Networks (BSN)*, (New York: IEEE), 79–82.

[B31] Keogh E. Ratanamahatana C. A. (2005). Exact indexing of dynamic time warping. *Knowl. inform. Syst.* 7 358–386. 10.1007/s10115-004-0154-9

[B32] Kirschbaum C. Hellhammer D. H. (1994). Salivary cortisol in psychoneuroendocrine research: recent developments and applications. *Psychoneuroendocrinology*, 19 313–333. 10.1016/0306-4530(94)90013-2 8047637

[B33] Kristal-Boneh E. Raifel M. Froom P. Ribak J. (1995). Heart rate variability in health and disease. *Scand. J. Work Environ. Health* 21 85–95. 7618063 10.5271/sjweh.15

[B34] Kucera P. Goldenberg Z. Kurca E. (2004). Sympathetic skin response: review of the method and its clinical use. *Bratisl. Lekars. Listy* 105 108–116. 15253529

[B35] Kudielka B. M. Schommer N. C. Hellhammer D. H. Kirschbaum C. (2004). Acute HPA axis responses, heart rate, and mood changes to psychosocial stress (TSST) in humans at different times of day. *Psychoneuroendocrinology*, 29 983–992. 10.1016/j.psyneuen.2003.08.009 15219648

[B36] Lazarus R.S. Folkman S. (1984). *Stress, Appraisal and Coping.* Springer: New York.

[B37] Lee Y. C. Winston F. K. (2016). Stress induction techniques in a driving simulator and reactions from newly licensed drivers. *Transp. Res. Part F* 42 44–55. 10.1016/j.trf.2016.06.019

[B38] Li K. Rüdiger H. Ziemssen T. (2019). Spectral analysis of heart rate variability: time window matters. *Front. Neurol.* 10:545. 10.3389/fneur.2019.00545 31191437 PMC6548839

[B39] Liao W. Zhang W. Zhu Z. Ji Q. Gray W. D. (2006). Toward a decision-theoretic framework for affect recognition and user assistance. *Int. J. Hum. Comput. Stud*. 64 847–873. 10.1016/j.ijhcs.2006.04.001

[B40] Litman T. (2019). *Autonomous Vehicle Implementation Predictions.* Canada: Victoria Transport Policy Institute.

[B41] Liu D. Ulrich M. (2014). *Listen to Your Heart: Stress Prediction Using Consumer Heart Rate Sensors.* URL: http://cs229.stanford.edu/proj2013/LiuUlrich-ListenToYourHeart-StressPredictionUsingConsumerHeartRateSensors.pdf.

[B42] Malik M. (1996). Heart rate variability: standards of measurement, physiological interpretation, and clinical use: task force of the European Society of Cardiology and the North American Society for Pacing and Electrophysiology. *Anna. Noninvas. Electrocardiol*. 1 151–181. 10.1111/j.1542-474x.1996.tb00275.x

[B43] Mantilla J. Oudre L. Barrois R. Vienne A. Ricard D. (2017). “Template-DTW based on inertial signals: Preliminary results for step characterization,” in *2017 39th Annual International Conference of the IEEE Engineering in Medicine and Biology Society (EMBC) 2267-2270)*, (New York: IEEE). 10.1109/EMBC.2017.8037307 29060349

[B44] Matthews G. (2001). *A Transactional Model of Driver Stress.* Florida: University of Central Florida, 133–163

[B45] Matthews G. Reinerman-Jones L. E. Barber D. J. Abich J. IV (2015). The psychometrics of mental workload: multiple measures are sensitive but divergent. *Hum. Fact*. 57 125–143. 10.1177/0018720814539505 25790574

[B46] May J. G. Kennedy R. S. Williams M. C. Dunlap W. P. Brannan J. R. (1990). Eye movement indices of mental workload. *Acta Psychol*. 75 75–89. 10.1016/0001-6918(90)90067-p 2260494

[B47] McCraty R. Atkinson M. Tiller W. A. Rein G. Watkins A. D. (1995). The effects of emotions on short-term power spectrum analysis of heart rate variability. *Am. J. Cardiol*. 76 1089–1093. 10.1016/s0002-9149(99)80309-9 7484873

[B48] McEwen B. S. (2000). Definitions and concepts of stress. *Encycl. Stress* 3 508–509.

[B49] McGrath J. E. (1976). Stress and behavior in organizations. *Handb. Industr. Organiz. Psychol.* 1351:1396.

[B50] Meyer K. Rasch T. Schnotz W. (2010). Effects of animation’s speed of presentation on perceptual processing and learning. *Learn. Instruct*. 20 136–145. 10.1016/j.learninstruc.2009.02.016

[B51] Milstein N. Gordon I. (2020). Validating Measures of Electrodermal Activity and Heart Rate Variability Derived From the Empatica E4 Utilized in Research Settings That Involve Interactive Dyadic States. *Front. Behav. Neurosci*. 14:148. 10.3389/fnbeh.2020.00148 33013337 PMC7461886

[B52] Min B. C. Chung S. C. Park S. J. Kim C. J. Sim M. K. Sakamoto K. (2002). Autonomic responses of young passengers contingent to the speed and driving mode of a vehicle. *Int. J. Industr. Ergon.* 29 187–198. 10.1016/s0169-8141(01)00059-2

[B53] Model 50–2303-00 (2014). *Model 50–2303-00.* Gahannah, OH: Mindware Technologies.

[B54] Mühlbacher-Karrer S. Mosa A. H. Faller L. M. Ali M. Hamid R. Zangl H. Kyamakya K. (2017). A driver state detection system—Combining a capacitive hand detection sensor with physiological sensors. *IEEE Trans. Instrum. Measur*. 66 624–636. 10.1109/tim.2016.2640458

[B55] Mulder G. Moray N. (1979). *Mental Workload: Its Theory and Measurement.* New York: Springer.

[B56] Mulder L. J. M. Dijksterhuis C. Stuiver A. De Waard D. (2009). Cardiovascular state changes during performance of a simulated ambulance dispatchers’ task: potential use for adaptive support. *Appl. Ergon.* 40 965–977. 10.1016/j.apergo.2009.01.009 19249011

[B57] Nickel P. Nachreiner F. (2003). Sensitivity and diagnosticity of the 0.1-Hz component of heart rate variability as an indicator of mental workload. *Hum. Fact.* 45 575–590. 10.1518/hfes.45.4.575.27094 15055455

[B58] Palinko O. Kun A. L. Shyrokov A. Heeman P. (2010). “Estimating cognitive load using remote eye tracking in a driving simulator,” in *Proceedings of the 2010 Symposium on Eye-Tracking Research & Applications*, (New York: ACM), 41–144.

[B59] Peat J. Barton B. (2014). *Medical Statistics: A Guide to SPSS, Data Analysis and Critical Appraisal.* Hoboken: John Wiley & Sons.

[B60] Pedrotti M. Mirzaei M. A. Tedesco A. Chardonnet J. R. Mérienne F. Benedetto S. Baccino T. (2014). Automatic stress classification with pupil diameter analysis. *Int. J. Hum. Comput. Interact.* 30 220–236. 10.1080/10447318.2013.848320

[B61] Prasolenko O. Burko D. Halkin A. (2017). Galvanic skin response as a estimation method of the driver’s emotional state. *Am. J. Sci. Engin. Technol*. 2 50–56.

[B62] Qu W. Zhang Q. Zhao W. Zhang K. Ge Y. (2016). Validation of the driver stress inventory in china: relationship with dangerous driving behaviors. *Accid. Anal. Prev.* 87, 50–58. 10.1016/j.aap.2015.11.019 26642077

[B63] Rastgoo M. N. Nakisa B. Rakotonirainy A. Chandran V. Tjondronegoro D. (2019). A critical review of proactive detection of driver stress levels based on multimodal measurements. *ACM Comput. Surv.* 51:88.

[B64] Rezazadeh I. M. Firoozabadi M. Hu H. Golpayegani S. M. (2012). Co-adaptive and affective human-machine interface for improving training performances of virtual myoelectric forearm prosthesis. *IEEE Trans. Affect. Comput*. 3 285–297. 10.1109/t-affc.2012.3

[B65] Rohleder N. Wolf J. M. Maldonado E. F. Kirschbaum C. (2006). The psychosocial stress-induced increase in salivary alpha-amylase is independent of saliva flowrate. *Psychophysiology* 43 645–652 10.1111/j.1469-8986.2006.00457.x 17076822

[B66] Rosenthal R. (1991). *Meta-Analytic Procedures for Social Research*, Canada: Sage Publications.

[B67] Ross T. Burnett G. (2001). Evaluating the human–machine interface to vehicle navigation systems as an example of ubiquitous computing. *Int. J. Hum. Comput. Stud.* 55 661–674. 10.1006/ijhc.2001.0495

[B68] Saxena N. (2017). *Modelling the Effect of the Number of Stop-&-gos on the Route Choice Behaviour of Car Drivers*, Ph D. thesis, (Sydney: The University of New South Wales).

[B69] Segerstrom S. C. Miller G. E. (2004). Psychological stress and the human immune system: a meta-analytic study of 30 years of inquiry. *Psychol. Bull*. 130:601. 10.1037/0033-2909.130.4.601 15250815 PMC1361287

[B70] Senin P. (2008). *Dynamic time warping algorithm review.* Honolulu, USA: Information and Computer Science Department University, 855 1–23.

[B71] Staal M. A. (2004). *Stress, Cognition, and Human Performance: A Literature Review and Conceptual Framework.* California: Ames Research Center.

[B72] Sugiono S. Widhayanuriyawan D. Andriani D. P. Prasetya R. P. (2019). Investigating the Influence of Distance between Cars on the Driver Psychophysiology during Braking Using Eeg: a Case Study on Driving in Indonesia. *Acta Neuropsychol*. 17 329–339. 10.5604/01.3001.0013.6189

[B73] Sztajzel J. (2004). Heart rate variability: a noninvasive electrocardiographic method to measure the autonomic nervous system. *Swiss Med. Weekly* 134 514–522. 10.4414/smw.2004.10321 15517504

[B74] Tomer E. Lupu T. Golan L. Wagner M. Braw Y. (2018). Eye tracking as a mean to detect feigned cognitive impairment in the Word Memory Test. *Appl. Neuropsychol.* 27 49–61 10.1080/23279095.2018.1480483 30183408

[B75] Van Roon A. M. Mulder L. J. Althaus M. Mulder G. (2004). Introducing a baroreflex model for studying cardiovascular effects of mental workload. *Psychophysiology* 41 961–981. 10.1111/j.1469-8986.2004.00251.x 15563349

[B76] Vuksanović V. Gal V. (2007). Heart rate variability in mental stress aloud. *Med. Engin. Phys.* 29 344–349. 10.1016/j.medengphy.2006.05.011 16807051

[B77] Wagner M. Sahar Y. Elbaum T. Botzer A. Berliner E. (2015). Grip Force as a Measure of Stress in Aviation. *Int. J. Aviat. Psychol*. 25 157–170. 10.1080/10508414.2015.1162632

[B78] Wahlström J. Hagberg M. Johnson P. Svensson J. Rempel D. (2002). Influence of time pressure and verbal provocation on physiological and psychological reactions during work with a computer mouse. *Eur. J. Appl. Physiol*. 87 257–263. 10.1007/s00421-002-0611-7 12111287

[B79] Wang X. Ding X. Su S. Li Z. Riese H. Thayer J. F. Snieder H. (2009). Genetic influences on heart rate variability at rest and during stress. *Psychophysiology* 46 458–465. 10.1111/j.1469-8986.2009.00793.x 19226307 PMC3713464

[B80] Wiberg H. Nilsson E. Lindén P. Svanberg B. Poom L. (2015). Physiological responses related to moderate mental load during car driving in field conditions. *Biol. Psychol*. 108 115–125. 10.1016/j.biopsycho.2015.03.017 25857673

[B81] Yamaguchi M. Wakasugi J. Sakakima J. (2006). “Evaluation of driver stress using biomarker in motor-vehicle driving simulator.” in *2006 International Conference of the IEEE Engineering in Medicine and Biology Society*, (New York: IEEE), 1834–1837. 10.1109/IEMBS.2006.260001 17945672

[B82] Yerkes R. M. Dodson J. D. (1908). The relation of strength of stimulus to rapidity of habit formation. *J. Comp. Neurol. Psychol*. 18 459–482. 10.1002/cne.920180503

[B83] Zak Y. Parmet Y. Oron-Gilad T. (2020). “Subjective Workload Assessment Technique (SWAT) in Real Time: Affordable Methodology to Continuously Assess Human Operators’ Workload,”in *2020 IEEE International Conference on Systems, Man, and Cybernetics (SMC)*, (New York: IEEE), 2687–2694.

[B84] Zhai J. Barreto A. (2006). Stress detection in computer users based on digital signal processing of noninvasive physiological variables. *Proc. IEEE Annu. Int. Conf*. 2006 1355–1358. 10.1109/IEMBS.2006.259421 17946041

[B85] Zontone P. Affanni A. Bernardini R. Del Linz L. Piras A. Rinaldo R. (2020). Stress evaluation in simulated autonomous and manual driving through the analysis of skin potential response and electrocardiogram signals. *Sensors* 20:2494. 10.3390/s20092494 32354062 PMC7249664

